# The NHS England Jewish BRCA Testing Programme: overview after first year of implementation (2023–2024)

**DOI:** 10.1136/jmg-2024-110390

**Published:** 2024-12-22

**Authors:** Bethany Torr, Nicola Bell, Ruth McCarthy, Monica Hamill, Joshua Nolan, Sudeekshna Muralidharan, Charlotte Andrews, Mikel Valganon-Petrizan, Yasmin Clinch, Suzanne MacMahon, Alison Morilla, Angela George, Paul Ryves, Pooja Dasani, Moses Adegoroye, Helene Schlecht, George J Burghel, Wendy Ornadel, Nicole Gordon, Lisa Steele, Susana Lukic, Emily Watts, D Gareth Evans, Ranjit Manchanda, Clare Turnbull

**Affiliations:** 1 Division of Genetics and Epidemiology, The Institute of Cancer Research, London, UK; 2 Cancer Genetics, The Royal Marsden NHS Foundation Trust, London, UK; 3 North Thames Genomics Laboratory Hub, The Royal Marsden NHS Foundation Trust, London, UK; 4 North Thames Genomics Medicine Service Alliance, Great Ormond Street Hospital for Children NHS Foundation Trust, London, UK; 5 North West Genomic Laboratory Hub, Manchester University NHS Foundation Trust, Manchester, UK; 6 Division of Evolution, Infection, and Genomic Sciences, The University of Manchester, Manchester, UK; 7 Jnetics, London, UK; 8 Chai Cancer Care, London, UK; 9 Cancer Programme, NHS England, London, UK; 10 Nightingale and Genesis Breast Cancer Centre, Manchester University NHS Foundation Trust, Manchester, UK; 11 Wolfson Institute of Preventive Medicine, Queen Mary University of London, London, UK; 12 Department of Gynaecological Oncology, Barts Health NHS Trust, London, UK

**Keywords:** Genetics, Population, Public Health, Policy, Genetic Testing

## Abstract

**Summary:**

**Background:**

The NHS Jewish BRCA Testing Programme is offering germline *BRCA1* and *BRCA2* genetic testing to people with ≥1 Jewish grandparent. Who have an increased likelihood of having an Ashkenazi Jewish (AJ) founder germline pathogenic variant (gPV) compared with the general population.

Testing is offered via a self-referral, home-based saliva sampling pathway, supported by a genetic counsellor telephone helpline. A first-of-its-kind in the United Kingdom (UK) for population genetic testing, outside of research.

**Methods:**

We reviewed data from germline testing of 5389 people who registered during the soft-launch phase (January 2023–January 2024) and their families to observe trends in uptake and outcomes of testing.

**Results:**

Of the 5389 self-referrals, 4339 (80.5%) consented to testing. Of those with results returned, 2.3% (98/4,274) had a gPV (89.8% AJ founder and 10.2% non-AJ founder).

Notably, the detection rate was higher in men (42/790, 5.3%) compared with women (56/3484, 1.6%), with the proportion reporting known BRCA variants within the family prior to consent also significantly increased (13.1% compared with 9.2%, respectively).

**Conclusion:**

Overall detection rates of gPVs are similar to those reported elsewhere from Jewish population testing. The pathway, particularly for males, may attract uptake of testing by those previously aware of familial gPVs.

## Introduction

 People of Jewish ancestry have an increased carrier frequency of germline pathogenic variants (gPVs) in *BRCA1* and *BRCA2* compared with the UK general population frequency (up to ~1-in-40 in Ashkenazi Jewish (AJ) individuals; ~1-in-140 in Sephardi Jewish (SJ) individuals; ~1-in-250 in the general population) .**1,4** This increased frequency is a result of known founder variants, which account for ~90% of gPVs in this population. Accordingly, *BRCA* gPVs have been shown to contribute towards an increased proportion of breast and ovarian cancers (~10% and ~40%, respectively, in AJ individuals compared with ~3% and ~15% in the general population)^5^,^9^ .**^10^**

It has previously been estimated that, within the Greater London area where most people (53.6%) who identify as Jewish live, we have identified approximately ~11% of BRCA carriers (that is, based on an AJ population size of ~1 91 000, we have identified 550 out of a predicted 5000 carriers).^11 12^ Thus, we are missing a sizeable proportion of individuals with gPVs to whom targeted screening and risk-reducing interventions might be of benefit, such as salpingo-oophorectomy or bilateral mastectomy to reduce respectively ovarian and breast cancer risks.

While testing of BRCA genes in the general population is not currently feasible, those with Jewish ancestry represent a rational group in which to pilot pathways for population testing, on account of their higher frequency of founder PVs. Notably, in Israel, germline BRCA testing for all women with Jewish ancestry was launched in 2020.^13^ In England, until initiation of this programme, National Health Service (NHS) BRCA testing has only been available to individuals with Jewish ancestry via eligibility based on personal history of BRCA-related cancers at any age and/or a very strong and verified cancer family history, as delineated in the National Test Directory.^14^

UK-centred health economic analyses have demonstrated it to be cost-effective to offer BRCA gene testing to all individuals with at least one AJ grandparent as well as female SJ individuals, irrespective of family history.^15^ Additionally, studies demonstrate overall support from AJ individuals for population testing, although with several cited barriers, such as concerns about confidentiality, stigmatisation and marriage prospects .**^16 17^**

In 2022, with this supporting evidence and in line with the NHS objective to achieve 75% of cancers diagnosed at an early stage by 2028, the NHS England Cancer Programme established a strategic objective to launch a programme of genetic testing for people of Jewish ancestry. Thus, the NHS Jewish BRCA Testing Programme was established with a Clinical and Expert Advisory Group (EAG) through the North Thames Genomics Medicine Service Alliance as a 3-year programme to facilitate testing in this community. The data presented here are from the soft-launch phase of the pilot Programme.

## Patients and methods

### The programme and eligibility

The NHS Jewish BRCA Testing Programme offers germline genetic testing to all people (1) with at least one self-reported Jewish grandparent, (2) over the age of 18 years of age and (3) eligible for NHS care within England.

The Programme launched on 31 January 2023 with a soft-launch phase of 1 year to 31 January 2024, inclusive. During this period, advertising of the Programme was targeted to those engaged with two national community-specific organisations (Jnetics and Chai Cancer Care), via mailing lists, associated magazines, community education events and social media.

### The pathway

With agreement from the EAG, testing was offered in the community via a home-based pathway, with coordination and oversight from the Translational Cancer Genetics team from the Institute of Cancer Research and Royal Marsden NHS Foundation Trust (RMH).

The pathway incorporated features from the BRCA-DIRECT pathway (piloted and developed in the breast oncology setting) and the GCaPPS pathway (trialled for Jewish population testing).^18^,^20^ The pathway supported high-throughput testing by removing the necessity for people to attend genetic counselling appointments.

Instead, following self-referral online or over the telephone, a testing pack was sent in the post, containing:

- An information booklet, similar in educational content to pretest genetic counselling consultation.
- A consent form, based on the NHS Record of Discussion.**^21^**
- A saliva sampling kit.
- A personal details form, capturing information, such as: number of Jewish grandparents, how they heard about the Programme, and whether there were any known BRCA carriers within the family.

Access to genetic counsellor (GC) or administrative support was available via a telephone helpline (available 9:00–17:00, Monday–Friday, with extended hours on Wednesdays) or email. All individuals reporting a known BRCA-carrier in the family were contacted by a GC prior to proceeding with testing, with requirement for completion of a full genetic counselling consultation.

Testing results were returned via the post, with GC telephone appointments arranged for all variants of uncertain significance (VUS) and positive results within one working week. Referrals were made into downstream services, including local clinical genetics for ongoing management and Very High-Risk Breast Screening services.

Cascade testing was offered via the Programme, with information shared among families via standard ‘to whom it may concern letters’ provided by the GC after the results appointment. Relatives were required to contact the telephone helpline for a consultation with a GC prior to receiving a cascade testing pack. Alternatively, they could attend their local clinical genetics service, following referral from their general practitioner.

### Genetic testing

DNA extraction from saliva samples was completed within a single laboratory at RMH, part of the North Thames Genomics Laboratory Hub (NTGLH). *BRCA1* and *BRCA2* sequencing of extracted DNA was performed at either the NTGLH RMH laboratory or at the North West GLH Manchester University Hospitals NHS Foundation Trust (MFT) laboratory.

Full sequence analysis of all coding exons for small variants in *BRCA1/BRCA2* was performed; where NGS analysis suggested a copy number variant, MLPA was also performed. Cascade testing reported on the Jewish founder variants (NM_007294.4(*BRCA1*)c.68_69del (p.Glu23fs) (historical name: 185delAG), NM_007294.4(*BRCA1*):c.5266dup (p.Gln1756fs) (historical name: 5382insC)), and NM_000059.4(*BRCA2*):c.5946del (p.Ser1982fs) (historical name: 6174delT)) and the familial variant, if different. Variants classified as pathogenic, likely pathogenic, or ‘hot’ VUS were reported.

### Data analysis

Data for this review were extracted on 12 July 2024. Analysis was completed in Stata, V.17, with χ^2^ Fisher’s exact tests conducted for two-by-two comparisons and two-sample Mann-Whitney U tests for differences between means.

## Results

### Participants and demographics

During the soft-launch, 5389 people self-referred for testing via the Programme.

The mean age (±SD) was 43.9 (±12.8) ranging from 18 to 89, and the majority were of female sex at birth (4090/5389 or 75.9%). The largest proportion of people reported to be of ‘Orthodox Traditional’ denomination (1718/5389, or 31.9%), with 894/5389 (16.6%) reporting to be unaffiliated. This information was missing for 1174/5389 (21.8%).

### Uptake of genetic testing

At point of data extraction, 1012/5389 (18.8%) had not returned their consent following three reminders and 38/5389 (0.6%) actively declined testing.

**Figure 1:**
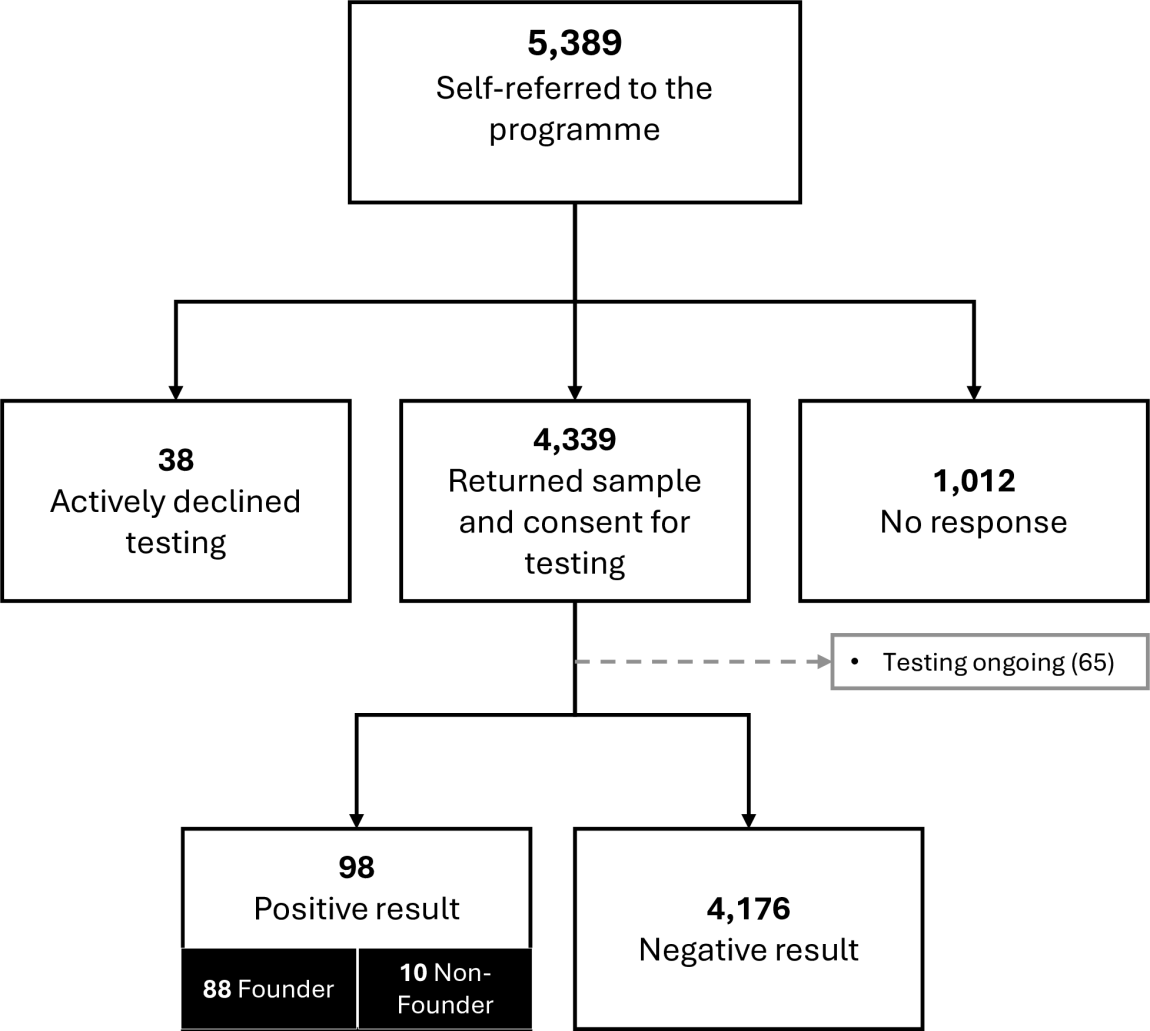
Participant progression during the Jewish BRCA Testing Programme soft-launch phase (31 January 2023 to 31 January 2024, inclusive).

4339/5,389 (80.5%) consented to testing (figure 1). Of these, among the 3532/4339 (81.4%) women, the mean age (±SD) was 43.7 (±12.4) ranging from 18 to 84, and among the 807/4339 (18.6%) men, the mean age was 47.4 (±15.0) ranging from 18 to 89.

### Access into the Programme

Information about how people had heard about the Programme was captured at point of consent. The main methods reported were: ‘friend or colleague’ (1547/4339, or 35.7%), ‘family member’ (1164/4339, or 26.9%) and ‘Jewish community leader or organisation’ (530/4339 or 12.2%). Participants could select more than one option. This information was missing for 36/4339 (0.8%) individuals consenting to testing.

### Testing and results

At point of data extraction, testing was still underway for 65/4339 (1.5%) participants. For those in whom testing had been completed, the failure rate on saliva samples was 0.3% (14/4288), incorporating 14 second samples which were attained and results reported for all failed primary samples. No blood tests were required.

For those in whom testing had been completed, the gPV pickup rate was 2.3% (98/4,274). No VUS were reported. Of the gPVs identified, 88/98 (89.8%) identified were one of the three Jewish founder variants (*BRCA1* (c.68_69del; p.Glu23fs), n=25, *BRCA1* c.5266dup; p.Gln1756fs n=3 and *BRCA2* (c.5946del; p.Ser1982fs) n=60).

#### Jewish ancestry

The pickup rate in those reporting four Jewish grandparents was 2.6% (86/3,303). Numbers were low for participants reporting one, two or three Jewish grandparent/s with 7/263, 3/541, 2/155 PVs detected. Information regarding grandparental ancestry was missing for 12/4274 people who had received results.

#### Known BRCA carriers within families ahead of testing

Of those in whom testing had been completed, 277 individuals reported a known BRCA carrier within the family ahead of testing. In regard of their closest BRCA-carrier relative, 126 reported ≥1 first-degree relative (FDRs)/s, 71 reported ≥1 second-degree relative/s (SDRs) (but no FDRs), and 80 reported ≥1 third-degree or ‘other’ relative/s (but no FDRs or SDRs).

Of the gPVs identified, 38/98 (38.8%) were in those reporting a known FDR BRCA carrier (a pickup rate of 30.2% (38/126)). Of those who did not report a known FDR BRCA carrier, the pickup rate was 1.4% (60/4148).

#### Sex at birth

The pickup rate varied significantly between male and female participants; 5.3% (42/790) in men and 1.6% (56/3,484) in women (p<0.001).

Reports of a known BRCA-carrier relative ahead of testing completion also differed significantly between male and female participants (70/790 (8.9%) compared with 207/3484 (5.9%), respectively (p=0.001)); with observed differences in proportion of the closest relative being first-degree, second-degree and third-degree/‘other’ reported by men being (53/70 (75.7%), 5/70 (7.1%) and 12/70 (17.1%), respectively) compared with women (73/207 (35.3%), 66/207 (31.9%) and 68/207 (32.9%), respectively).

Of those identified to have a gPV, 22/42 men (52.4%) and 16/56 (28.6%) women reported a known FDR BRCA carrier ahead of testing, with the observed pickup rate in those reporting a known FDR BRCA carrier being 22/53 (41.5%) and 16/73 (21.9%), respectively.

### Familial testing

At point of data extraction, 47/49 individuals contacting the service about cascade testing had consented to genetic testing. A pickup rate from completed cascade tests of 44.7% was observed. Cascade tests completed per family varied from 0 to 5 (mean (±SD), 0.5 (±1.1)), with the median (IQR) follow-up time since the proband received results being 8 (5 to 10) months. Within 21.6% of families with a positive result, two or more individuals had independently self-referred to the Programme (thus are not counted as ‘cascade tests’) and were later identified to be related.

### Hotline use

Over the 1-year period, 827 hotline calls, totalling 5200 min (86.7 hours) were recorded. These hotline calls included all calls made by people to the service (ie, excluded contact initiated by Programme administrators or GCs). Of which, 692/827 (83.7%) were from 480 known participants (372/480 (77.5%) women and 96/480 (20.0%) men), ranging from 1 to 7 calls per person (median (IQR): 1 (1–2). 135/827 (16.3%) call records could not be linked to an individual meaning that the caller either did not wish to provide their details, or had not self-referred at the time of the call. Where recorded, most calls (803/819, or 98.0%) were made within working hours (9:00–17:00 Monday-to-Friday).

Most hotline calls were administrative (557/827, 67.4%). However, calls requiring a GC represented the greatest proportion of time (3263/5200 min, 62.8%), with a significant difference observed in average (mean±SD) length of calls requiring a GC compared for administrative calls (12.3 (±11.1) vs 3.5 (±2.0), respectively (p<0.001).

## Discussion

We here present outcomes from the first year of the NHS Jewish BRCA Testing Programme launched in England in 2023. The first population-based, unselected germline genetic testing programme implemented to date.

The pickup rate in index cases was 2.3% (1-in-43) and in line with previously reported figures from Jewish population testing for those with four (Ashkenazi) Jewish grandparents, for which most (76.1%) participants here reported. The identification of ~10% non-founder variants supports the rationale for offering full *BRCA1* and *BRCA2* testing, which was previously reported by Desai *et al*^22^, who found similar pickup rates of founder (89.9%) variants in those with full AJ ancestry within direct-to-consumer testing.^22^ Taken together, these points demonstrate that the Programme was effectively targeted at and taken up by the intended community using self-reported ancestry.

We observed a significant difference in the pickup rate in biological men (5.3%) compared with women (1.6%). This would appear to have been driven, at least in part, by a greater proportion of male participants being correctly aware of known BRCA carrier/s within FDRs ahead of self-referral. This may reflect differences in health-seeking behaviours and indicates that this NHS-funded, easy-access, home-based pathway may encourage uptake of testing by those not previously seeking cascade testing. Alternatively, there may be perceived lack of relevance of BRCA testing to men; thus the increased pickup rate may be a result of communications by the engagement partners around male BRCA risks and benefits of testing. Nonetheless, given the overall lower levels of uptake in men compared with women, it is important that these factors are considered, and measures introduced that seek to address the overall imbalance in uptake.

We did not collect information on personal or family history of cancer, so are unable to understand the extent to which this influenced individuals’ prior likelihood of having a gPV or assess whether this contributed to the observed differences. However, given ‘advertising’ during this period was driven towards those engaged with two Jewish community-specific patient groups related to genetics (Jnetics) and cancer (Chai), it is probable that people were likely to (1) be affiliated with the Jewish community and (2) have personal/family history of cancer and/or awareness of BRCA genes. On full rollout of the Programme, more generalised communications are being deployed, aimed at reaching unaffiliated or non-practising individuals of Jewish ancestry. Close monitoring will therefore be required, given the model of relying on self-reported Jewish ancestry and self-referral.

Most participants (62.6%) reportedly heard about the Programme via family, friends or colleagues, demonstrating the value of word-of-mouth in disseminating information among eligible people.

This is an important factor and particularly encouraging for facilitating cascading testing among family members of those identified to be positive. Notably, numbers at this stage appear to be low for cascade testing (mean of 0.5 individuals per positive patient). Based on the previous evidence, we would expect this to increase over time given current median follow-up time is relatively short (8 months).^23^ Furthermore, upon review of family pedigrees at results appointments, we were able to identify where multiple relatives had registered for the testing at the same time, thus being considered as separate individuals and not being accounted for in the ‘cascade’ figures. Finally, some relatives may have chosen to be tested via their local Clinical Genetics service, numbers of which we are unable to capture. Facilitating uptake of cascade testing will be important to maximise opportunity for identifying BRCA carriers via the Programme.

High-throughput sample-handling pathways have proven essential for delivery of this Programme, with >15 000 additional individuals registered in the 3 months after formal launch (1 February 2024) and an overall target to deliver 30 000 tests (approximately 10% of those identifying as Jewish in England) over the full 3-year pilot programme.^11^ At the NTGLH laboratory, automated workflows have been implemented to optimise throughput as well as yield and quality of DNA extracted from saliva samples.^24^ Sequence analysis failed in only 0.3% of saliva samples, affirming as viable and effective the use of saliva as the primary sample type.

Overall, early evidence is demonstrating effectiveness and suitability of the approach implemented in England for testing of the Jewish community, including the clinical pathway of self-referral, home-based testing utilising saliva-sampling, support via the GC hotline, and full sequence analysis of the *BRCA1* and *BRCA2* genes. Given this is the first NHS population germline genetic testing programme delivered in England, key areas planned for full evaluation include understanding participant satisfaction with the pathway, impact on downstream services and long-term outcomes.
